# Which experimental systems should we use for human microbiome science?

**DOI:** 10.1371/journal.pbio.2005245

**Published:** 2018-03-19

**Authors:** Angela E. Douglas

**Affiliations:** Department of Entomology and Department of Molecular Biology and Genetics, Cornell University, Ithaca, New York, United States of America

## Abstract

Microbiome science is revealing that the phenotype and health of animals, including humans, depend on the sustained function of their resident microorganisms. In this essay, I argue for thoughtful choice of model systems for human microbiome science. A greater variety of experimental systems, including wider use of invertebrate models, would benefit biomedical research, while systems ill-suited to experimental and genetic manipulation can be used to address very limited sets of scientific questions. Microbiome science benefits from the coordinated use of multiple systems, which is facilitated by networks of researchers with expertise in different experimental systems.

One of the great biological success stories over the last decade is microbiome science, including a growing understanding of the impact of resident microorganisms on human health. We now have irrefutable evidence that microorganisms are key players that shape many functions of humans and other animals, from metabolism to immunity and behavior [1]. Furthermore, our growing understanding of and capacity to manipulate the resident microorganisms are already translating into real-world benefits. For example, a microbial therapy reliably eliminates life-threatening *Clostridium difficile* infections in people [2,3], and there are realistic prospects to suppress mosquito transmission of dengue virus by the large-scale release of mosquitoes modified to bear a bacterium *Wolbachia* that confers vector incompetence [4,5].

Underlying the recent successes and excellent prospects for the discipline of microbiome science, there are, however, differences of opinion, especially concerning the utility of different experimental systems to advance our understanding and application of microbiomes for human health. The appropriate choice of study system is vitally important for everyone from individual researchers and their institutions to national and international funding organizations. The choices facing biomedical applications of microbiome science have many parallels with other disciplines in the life sciences as advancing technologies in molecular biology, cell biology, and microbiology enable us to answer new questions and solve previously intractable problems.

Two linked factors play an important role in the appropriate choice of experimental systems for human microbiome research: history and purpose. Let us start with history. For many, the discipline of microbiome science burst onto the scientific scene some 10 to 15 years ago, made possible by breakthroughs in sequencing technologies [6] that enabled us to identify and study the function of microorganisms in situ, i.e., in their natural communities without isolation into clonal cultures [7,8]. For the first time, it became practicable to study the complex communities associated with humans and other animals. The power of this new science was swiftly recognized by the biomedical community, harnessing microbiome science as a route for improved understanding and treatment of human disease, especially chronic diseases associated with dysfunction of metabolism and immunity, e.g., obesity, inflammatory bowel disease [9–12]. This potent combination of history and purpose identified the human as the ideal biological system for study. For ethical reasons, human studies generally yield correlations between microbial traits (composition and function) and phenotype (including disease state), requiring experimentation on other systems to determine causality and mechanism (Fig 1). To a very large extent, a single traditional model, the laboratory mouse, has fulfilled the important role as experimental model of host–microbe interactions in humans [13].

**Fig 1:**
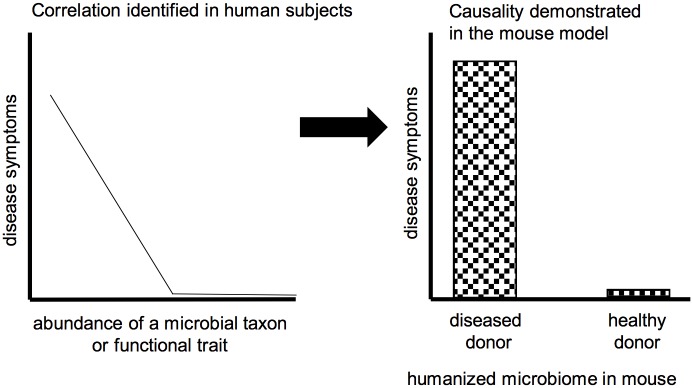
The laboratory mouse model is widely used to demonstrate causality of correlations between the microbiome and human disease. (A) Analysis of many humans reveals a negative association between the severity of disease symptoms and an index of the microbiome, e.g., abundance of a specific taxon or functional trait. (B) “Humanized” mice (i.e., mice colonized with microbiome samples from humans) are used to infer a causal role of the microbiome in the human disease: mice display disease symptoms when colonized with microbiome samples from diseased, but not healthy, humans. This figure is a generalized illustration and is not intended to be representative of any specific disease or microbiome dataset.

So where is the problem? It is that many biomedical researchers and their funders are neglecting an important strand of our history and, consequently, may be selling themselves short. The “new” discipline of microbiome science did not enter an empty playing field. Instead, and to its great benefit, microbiome science builds on more than a century of research on microorganisms in healthy animals: the discipline of symbiosis [14]. Eukaryotic organisms—from the first unicellular eukaryotes to complex, multicellular groups (including animals)—have repeatedly entered into alliances with microorganisms, enabling them to exploit otherwise unavailable habitats and, for animals, unsuitable diets [15]. As befits a mature discipline, the goals in symbiosis research are diverse. Some questions are driven by ecological and evolutionary considerations. How do the presence and composition of its microbiota influence the ecological fit and the fitness of an individual host and—at larger scales—the structure of ecological communities and evolutionary trajectory of host lineages [16–20]? Other questions are mechanistic: what is the molecular basis of microbial persistence in their host, including the patterns of host–microbe signal and nutrient exchange, and how are these often coevolved traits influenced by the mode of transmission and evolutionary age of the association [21–23]? Over many decades, the associations used to address these questions have been selected for their microbiological simplicity, ideally a single microbial taxon that is restricted to a specific host organ and is morphologically conspicuous (e.g., the pigmented algal symbionts in corals, the luminescent bacteria in squid). The microbiology of the traditional animal models does not meet these criteria, and symbiosis researchers have been adept at identifying and developing alternative systems to answer their questions. This diversity of system and purpose has led to the opposite mindset from the biomedical microbiome scientist. For the symbiosis researcher, there is an inherent value in diversity of systems. Not only are different biological systems suitable for different questions, but multiple systems used to address the same question are predicted to yield a richer understanding of any one topic.

For the sake of clarity and brevity, the preceding paragraphs portray the assumptions and expectations in our discipline as a simple dichotomy between the “mature” field of symbiosis research and the “new” field of biomedical microbiome science. Although this is an accurate reflection of the view of some colleagues, I believe that the perspective of many practitioners in the discipline is more nuanced. There is a growing sense that the application of microbiome science for human health would be better served by the use of a greater range of experimental models by the biomedical community and, equally, that fundamental discovery will be facilitated by a greater focus on systems that are experimentally tractable and amenable to the latest molecular techniques. How can we achieve these beneficial outcomes?

A first-order question for the future of microbiome science is the utility of the traditional animal models that have been the basis for fundamental biological discoveries over many years (Fig 2A). These are the animal systems that are maintained indefinitely under laboratory conditions; are amenable to genetic manipulation; have genetically defined wild-type strains, enabling the phenotype of mutations to be investigated against a common genetic background; and have attracted the critical mass of investigators needed to develop common tools and structures for sharing resources and data, with dissemination via stock centers, databases, etc. These models are also tractable to the key procedures in microbiome science—to generate microbiologically sterile (“germ-free”) animals that can be recolonized with a standardized microbiota (Fig 2B) [24–29]—and their value is being enhanced further by advances in experimental manipulation of the microbial partners. In particular, protocols for enumeration, cultivation, and genetic manipulation of key members of the microbiome are either in place or the subject of intensive method development [30–33]. Nevertheless, some of these traditional models are not currently being used to their full capacity in microbiome science.

**Fig 2:**
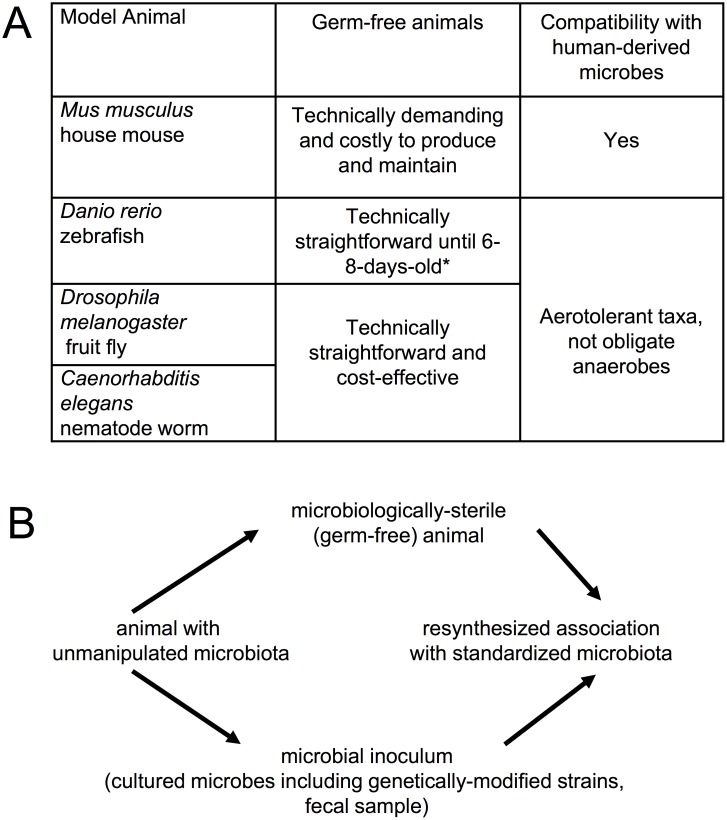
The traditional animal models are amenable to microbiome research. (A) The advantages and limitations of traditional models for microbiome research. *Zebrafish older than 6–8 days require feeding, which is technically demanding under strictly sterile conditions [27]. (B) The key microbiological manipulations required for experimental investigation of microbiome function.

Many microbiome scientists focused on human health make extensive use of the experimental protocol illustrated in Fig 1 to identify a causal role of the microbiome in human disease. This approach requires that the animal model be colonized by human-derived microbes and respond to them in the same way as humans. To a certain extent, the laboratory mouse meets these expectations. Nevertheless, great caution is needed to extrapolate mouse data back to the human. There are major anatomical differences between the gastrointestinal (GI) tract of the mouse and human, and the laboratory mouse is highly inbred and maintained under pathogen-free conditions that bear no resemblance to the human condition. The use of mice bearing human-derived microbes is particularly problematic because an appreciable proportion of human-associated gut microorganisms fail to colonize the mouse gut, and those microbial taxa that do colonize fail to induce some responses elicited by the native mouse microbiota [34–36]. Two responses to these limitations of the mouse model are appropriate. The first is to investigate microbial interactions with human cells grown in vitro as sheets of gut epithelium or as GI-tract organoids, and we are witnessing rapid progress with these models [13,37,38]. The second response builds on insights from symbiosis research, specifically that the interactions between the human and its microbiota are the product of a long evolutionary history of interactions between animals and benign or beneficial microorganisms. The eukaryotic forbears of animals lived in a world that had been inhabited by bacteria for billions of years, with the expectation that—as for modern unicellular eukaryotes and basal animals—the ancestral animals were colonized by a diversity of microorganisms, with which they interacted [39]. Consequently, we should expect (and indeed, are finding) conserved genetic and cellular mechanisms underlying interactions between various animals and their resident microbiota [1]. The implication is that fundamental discoveries in microbiome science can be made using the most tractable animal systems, including nonmammal vertebrates such as the zebrafish and invertebrates such as *Drosophila* and *Caenorhabditis elegans* (Fig 2A). The human may, in a perfect world, be the best model for the human—but arguably, we will obtain a better understanding of the human–microbiome interactions faster and more cost-effectively by more extensive integration of “simple” animal models into the collective endeavor.

Other practitioners in the discipline are wary of the traditional animal models because of the perception that the microbiota of these models is unnatural as a result of laboratory cultivation. There is some truth in these concerns. The gut microbiota of the laboratory mouse is different from wild conspecifics [40], taxonomically indistinguishable bacteria in wild and laboratory *Drosophila* differ in functional gene content [41], and the oft-repeated claim that *C*. *elegans* has no microbiota arises from a standard laboratory practice that maintains this species in a microbiologically sterile condition apart from a single *Escherichia coli* strain used as food [25]. These constraints can, however, be overcome by thoughtful experimental design. We are already witnessing evidence-based discussion about the wisdom of maintaining laboratory mouse cultures under intensely hygienic conditions [42,43], and research on *C*. *elegans* reunited with its natural microbiome is yielding new insights into the immune function of this model [25].

What about the systems that are being used to study animal–microbe interactions but lack the in-depth infrastructure available to the traditional models? A number of systems used by relatively small communities of researchers have yielded major insights of general significance. I cite a few among many examples here: hydra for the role of the microbiota in orchestrating peristalsis of the gut epithelium [44], squid for integration of microbial function into the circadian rhythm of the host [45], the aphid for coevolved metabolite exchange [46], and the killifish for microbial determinants of lifespan [47]. For these systems, the future is bright because of the progressive democratization of technologies. Tools and resources that once demanded an army of researchers with funds to match can now be developed by small but well-integrated teams. For your favorite organism, you can sequence its genome or modify its genes and their expression by a growing list of genetic technologies, including RNA interference (RNAi), genome editing, and the use (or set up) of publicly available online databases to share information and resources. An early-career researcher entering the discipline is well-advised to consider whether their preferred system has, or has good prospects of, these resources for the host or microbial partners.

My conclusion is somewhat akin to Goldilocks’ porridge: neither too few nor too many. Let us not invest our resources in a narrow set of systems, especially when other systems (often less familiar to the biomedical community) would yield results faster and more economically. And let us be cautious of systems that are ill-suited to experimental and genetic manipulation. The middle ground does, however, create a substantial constraint for the individual research group. The use of any biological system has large front-end costs, including the financial cost of setting up facilities and the opportunity cost of developing the expertise. These costs reduce our flexibility. It is difficult for researchers to use multiple systems in one study, for example, to first investigate a problem in a “simple” system and then to test for its relevance to mammals (but see [48]); and it is difficult for a single researcher to switch between different systems as their research questions change over time. I believe that it is important for the future of our discipline to mitigate this constraint. One powerful solution is already starting to happen: networks of researchers who can share access to multiple different systems (traditional models, nontraditional models, in vitro systems, and human research) and the associated expertise. These networks can be (partly or entirely) colocated as centers at one (or several) institution(s). Combined with a greater awareness of the opportunities available from different biological systems, these institutional innovations will make it easier for us all to choose the best biological model, and to use it well.

## Abbreviations

GI: gastrointestinal
RNAi: RNA interference
